# Population spatialization at building scale based on residential population index—A case study of Qingdao city

**DOI:** 10.1371/journal.pone.0269100

**Published:** 2022-05-26

**Authors:** Zhen Mao, Haifeng Han, Heng Zhang, Bo Ai

**Affiliations:** 1 College of Geodesy and Geomatics, Shandong University of Science and Technology, Shandong, China; 2 Shandong Provincial Institute of Land Surveying and mapping, Shandong, China; China University of Geosciences, CHINA

## Abstract

The study of population spatialization has provided important basic data for urban planning, development, environment and other issues. With the development of urbanization, urban residential buildings are getting higher and higher, and the difference between urban and rural population density is getting larger and larger. At present, most population spatial studies adopt the grid scale, and the population in buildings is evenly divided into various grids, which will lead to the neglect of the population distribution in vertical space, and the authenticity is not strong. In order to improve the accuracy of the population distribution, this paper studied the spatial distribution of population at the building scale, combined the digital surface model (DSM) and the digital elevation model (DEM) to calculate the floor of buildings, and proposed a new index based on the total floor area of residential buildings, called residential population index (RPI). RPI is directly related to the number of people a building can accommodate, so it can effectively estimate the population of both urban and rural areas even if the structure of urban and rural buildings is very different. In addition, this paper combined remote sensing monitoring data with geographic big data and adopted principal component regression (PCR) method to construct RPI prediction model to obtain building-scale population distribution data of Qingdao in 2018, providing ideas for population spatialization research. Through field sampling survey and overall assessment, the results were basically consistent with the actual residential situation. The average error with field survey samples is 14.5%. The R^2^ is 0.643 and the urbanization rate is 69.7%, which are all higher than WorldPop data set. Therefore, this method can reflect the specific distribution of urban resident population, enhance the heterogeneity and complexity of population distribution, and the estimated results have important reference significance for urban management, urban resource allocation, environmental protection and other fields.

## Introduction

Population distribution data, as the basic data that reflects social and development situation of a country or city [1, 2], is also one of the most important basic data in social and geographical research, being widely used in urban planning, social resource allocation, environmental protection and other fields [3, 4]. The existing population distribution data are largely dependent on demographic data, which are often collected step-by-stepwith administrative divisions as units, which not only take a long time to update and consume a lot of manpower, but also have low spatial and temporal resolution, making it difficult to express spatial distribution details. Therefore, it is necessary to integrate other data to explore the spatial distribution of population. This process of discrete census data based on other data is called population spatialization [5]. Population spatialization is one of the important methods for coupling population with other socio-economic, resource and environmental data. This is of great significance for improving the capacity of integrated management of population, resources and environment.

In the past few decades, geospatial information technology has developed rapidly, providing the possibility of more accurate estimation of population density. Scholars have explored methods for estimating population density by integrating various multi-source data. Some scholars believe that the models for simulating population distribution mainly include spatial autocorrelation model, spatial logistic regression model and density-independent matrix model [6]. Starting from the basic principle and development process of population spatialization, Bai et al. believe that population spatialization methods include urban geographic population density model, spatial interpolation method and statistical model based on remote sensing and geographic information system (GIS) [7]. Among them, the urban geographic population density model is not directly applied to the study of population spatial, but more generally to the population distribution [8]. Spatial interpolation method is mainly applied to the regional conversion of census data [9], which can improve the resolution of results. However, it is easily affected by the selection of original data and interpolation methods, and it is difficult to reflect the details of the real population distribution. Statistical modeling based on remote sensing and GIS can overcome the above difficulties, which is the focus of current research.

Although the population spatial research based on remote sensing and GIS can greatly improve the efficiency of simulating population distribution, most of the research results in recent years are presented by grids with the size of 130~1000m [10], the population living in high-rise buildings can only be scattered on the ground in a larger area, and there is no corresponding relations between population and residential buildings, so the vertical spatial distribution of population is not fully considered. In countries with diverse housing structures and high population density, such as China, there will be some deficiencies in such population spatial results.

As for the consideration of integrating multi-source data, many studies are based on land use/land cover (LULC) [10–13]. Although it has been proved that the population spatial method combined with land classification is very suitable for China’s population distribution, other data can be further integrated on this basis to improve the accuracy of results. Since NTL data can sensitively detect lights of different degrees and reflect the development trend of the city, such as economic development [14]. So there is a strong correlation between human activitiesand NTL, many studies have begun to adjust population spatial positioning results by combining NTLdata [15, 16]. Some scholars proposed using The Defense Meteorological Satellite Program Operational Linescan System (DMSP-OLS) and normalized dierence vegetation index (NDVI) data to study the human settlement index (HSI) of population distribution in Zhejiang Province, China [17]. However, few studies have been conducted on the spatialization of urban population based on DMSP-OLS data, because it has many obvious defects, especially the phenomenon of "oversaturation" [18, 19] and low spatial resolution (1km). The emergence of Suomi National Polar-Orbiting Partnership Visible Infrared Imaging Radiometer Suite (NPP-VIIRS) data overcomes the shortcomings of DMSP-OLS data, but its data [20, 21] have a lot of background noise and outliers, and the resolution of 500m still has some limitations in fine-scale studies of population spatial. In June 2018, Wuhan University designed and successfully launched the Luojia 1–01 satellite, which is the world’s first professional luminous remote sensing satellite. Its spatial resolution and quantitative level have been significantly improved. With a spatial resolution of about 130 m. Previous studies have shown that in simulating population distribution, the fitting effect of LuoJia 1–01 data is generally superior to DMSP/OLS and NPP-VIIRS data, which alleviates spillover effects to a certain extent [22–24]. The high resolution of NTL images brings new opportunities for the study of urban population spatial distribution. For example, due to the negative correlation between vegetation cover and population density, some scholars proposed the human settlement index (HSI) by combining NTL and enhanced vegetation index (EVI) to describe the spatial distribution of population [25]. In addition, some scholars combined remote-sensing big data to simulate population distribution, adding traffic or social media data, such as subway [26] and taxi [27] data, or integrating POI data [6]. The integration of remote sensing big data can enhance the spatial heterogeneity of population distribution to a certain extent and improve the accuracy of population spatialization, but the selection of influencing factors plays a decisive role, so factors affecting population distribution should be considered as fully as possible.

In view of the above problems, such as insufficient refinement of the results, lack of consideration of the vertical distribution of population, and insufficient synthesis of the selection of influencing factors, this paper makes the following optimization. We chose detailed and specific residential building data as the unit to simulate the resident population distribution, which improved the refinement of the simulation and enhanced the spatial heterogeneity of the population distribution. At the same time, we considered the height of residential buildings and established the residential population index (RPI) combining Digital Surface Model (DSM) and Digital Elevation Model (DEM), which can reflect the number of the resident population per unit floor area. When considering the factors affecting the RPI, we integrated the multi-source city geographic data, combined with the NTL, EVI and the point of interest (POI) data of various industries, comprehensively analyzed the impact of various factors on the RPI, and adopted the principal component regression (PCR) method to generate the RPI prediction model. The prediction model not only takes into account natural and social factors, inherits remote sensing big data, improves the prediction accuracy, but also reduces the prediction cost and makes the census more convenient. Finally, street boundaries and street resident population were used as control units to discuss the total population and test the accuracy of simulation. the error is lower than the WordPop dataset, which is the dataset with the highest estimated accuracy demonstrated by studies in the available grid population spatial distribution datasets (such as CNPOP, GPW, WorldPop, etc.) [6]. Therefore, the prediction model adopted in this paper has high practicability and reliability, and can reflect the distribution of permanent resident population in residential units. The data results will provide important help in policy implementation, disaster rescue, social research and other aspects, and further show that the integration of multi-source urban geographic data has strong application potential in population spatial research.

## Materials and methods

### Study area

**Fig 1:**
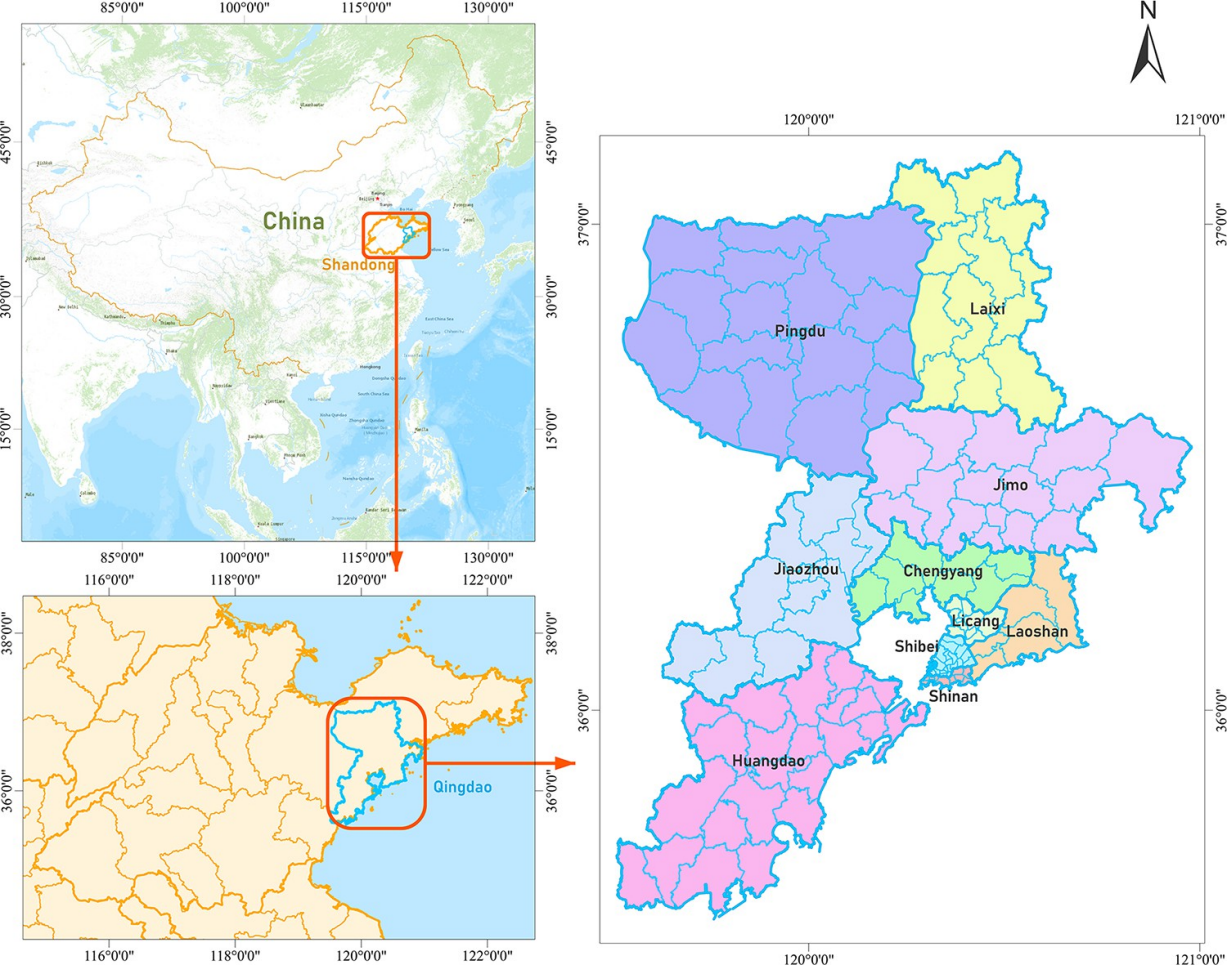
Qingdao geographical location map [30].

Qingdao is located in eastern China, southeast of Shandong Peninsula, bordering the Yellow Sea (Fig 1). It is a sub-provincial city of Shandong Province, a city specifically designated in the state plan, an important city along the east coast of China, and an international port city [28]. As of 2018, Qingdao has jurisdiction over 7 districts and 3 county-level cities, with a total area of 11,293.36 square kilometers, including 715.1 square kilometers of built-up area, and with a permanent population of 9.3948 million, among which 6.3525 million are permanent residents in urban areas [29].

As an important city along the east coast of China with a high level of urbanization development, the urbanization rate of Qingdao has reached 73.67%. However, as the built-up area of Qingdao is only 7.62% of the city’s area, population is highly concentrated in the urban area, and there is a huge gap between urban and rural population density. Therefore, taking Qingdao as the research area plays an important role in the population spatial research of cities with the high urbanization rate.

### Data sources

Table 1 presents information on the data used in this study. Due to different data sources, in order to eliminate the influence caused by different coordinate systems, all research data will adopt the China Geodetic Coordinate System 2000 as the projection coordinate system.

**Table 1:** Data sources table.

Name	Data content	Spatial resolution	Time	Data source
DSM	Digital Surface Model	2m	2018	The geographical monitoring data of Shandong Province.
DEM	Digital Elevation Model	2m	2018	The geographical monitoring data of Shandong Province.
NTL	Luojia 1–01 NTL data	130m	June to October 2018	The High Resolution Earth Observation System of Hubei Data and Application Center website. Availabe online: http://59.175.109.173:8888/app/login.html
EVI	MOD13Q1-EVI data	250m	June to October 2018	The Data Information Service Center of National Aeronautics and Space Admin-istration. Availabe online: https://ladsweb.modaps.eosdis.nasa.gov/
Basic geographic data	The district and county boundaries, township streets boundaries, main road data,building vector data and urban built-up area data of Qingdao	-	2018	The geographical monitoring data of Shandong Province.
Residential land data	The land mainly used for the housing base and its ancillary facilities for people’s living	-	2018	The Third National Land Survey started field work in 2018. Work Manual of the Third National Land Survey. Availabe online: http://www.mnr.gov.cn/zt/td/dscqggtdc/zl/201906/t20190604_2439983.html
POIs	There are 21 first-level industries, which are Restaurant, Hotel, Shopping, Living Services, Beauty, Scenic Spot, Entertainment, Exercise Fitness, Educational Training, Cultural Media, Medical Care, Automotive Service, Transportation Facilities, Finance, Real Estate, Company Enterprise, Government Organs, Entrance and Exit, Natural Features, Administrative Land-marks, Portal Address respectively	-	2018	Baidu map POI industry classification. Availabe online: https://lbsyun.baidu.com/index.php?title=lbscloud/poitags; Baidu Map LBS Cloud Service. Availabe online: http://lbsyun.baidu.com/index.php?title=lbscloud
Demographics	The data of permanent resident population of each subdistrict and township in Qingdao	-	2018	*Qingdao Yearbook 2019*. Availabe online: http://qdsq.qingdao.gov.cn/n15752132/n20546841/n32568999/index.html
Worldpop dataset	One of the most accurate data sets available in the world to estimate population distribution in the grid spatial population distribution data set	100m	2018	China’s Worldpop dataset. Availabe online: https://www.worldpop.org/geodata/summary?id=24924

### Methodology

**Fig 2:**
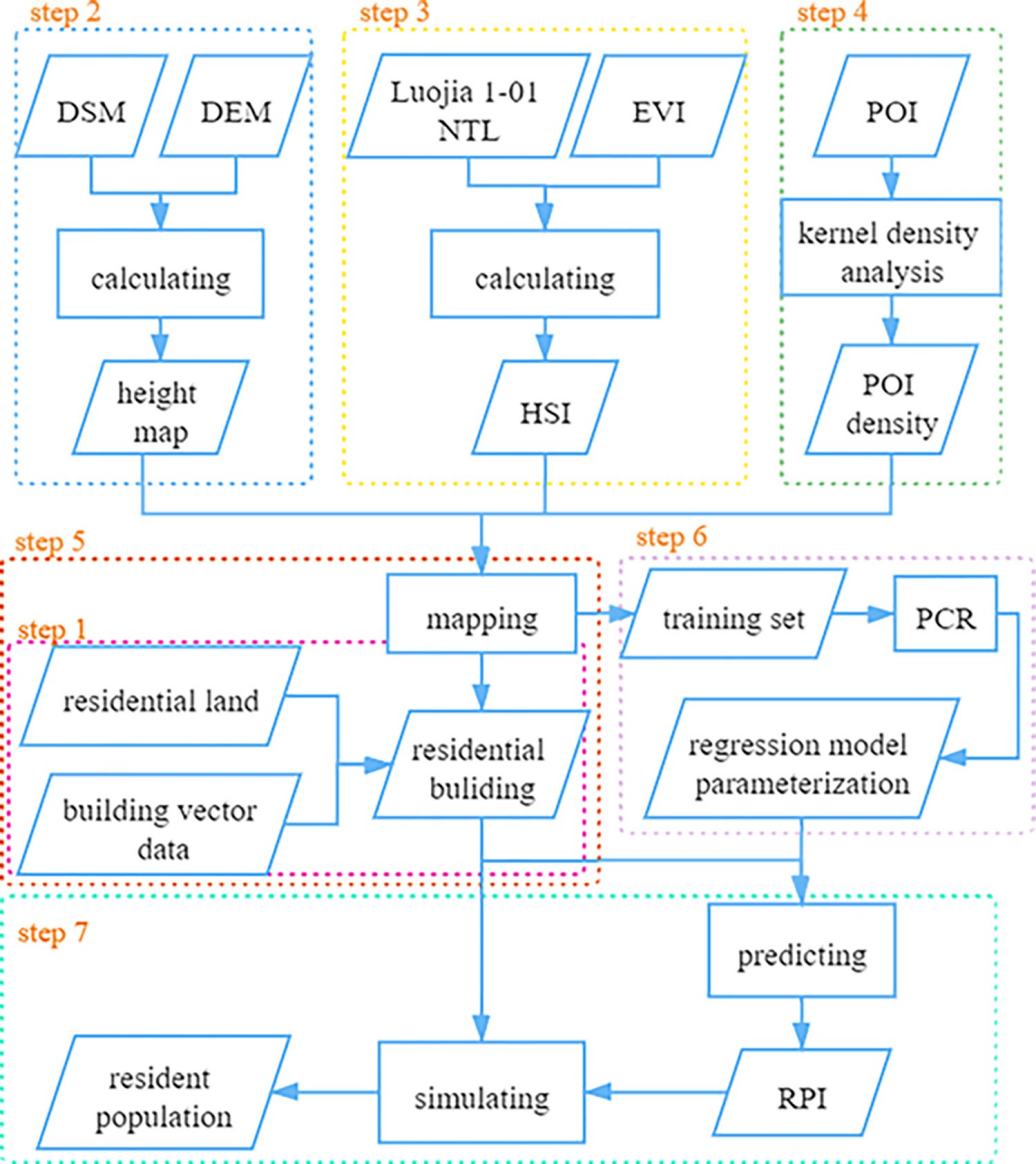
Flowchart of the residential population distribution model based on residential buildings.

In previous studies, population spatial results were often presented in raster format, which could not generate fine residential population data. As a basic database, it had significant limitations. In order to better assist the census work, as shown in the Fig 2, we specially used the data of residential buildings to study the distribution of resident population. Since, under normal circumstances, the permanent population lives in the residential buildings on the residential land, it is reasonable to use the range of the residential buildings to allocate the permanent population. But choosing the base area of residential buildings to estimate the population is not enough. With the deepening of urbanization, urban residential buildings are getting higher and higher. Combined with DSM and DEM, we calculated the number of floors of residential buildings, and obtained the total floor area through the base area of residential buildings, and proposed a new index, namely Residential Population Index (RPI), to represent the number of the resident population per unit floor area. According to the calculation method of RPI, it can be concluded that the size of the habitable area of a residential building has determined the upper limit of its population. Therefore, in the face of the differences between urban and rural building structures, RPI will not wrongly overestimate the population due to the large base area in rural areas, and can be applied to estimate the residential population of both urban and rural areas. To further reflect population distribution, the RPI also needs to be adjusted in combination with multi-source data. Since NTL is positively correlated with population distribution, it is often used as an indicator to reflect population distribution. Meanwhile, combined with EVI, which is negatively correlated with impervious water surface, HSI is used as the component affecting population distribution in this paper [15, 31, 32]. In order to improve the simulation accuracy, we also combined POI data, which reflects social perception, and use the kernel density values of various POIs as the distance between residential buildings and the intensive points of various industries, so as to explore the influence of various industries on the distribution of resident population. Combining all the influencing components, we predicted the RPI of residential buildings by Principal component regression (PCR) and finally simulated the residential resident population. This method can not only refine the population distribution, enhance the complexity and inhomogeneity of population distribution, but also integrate various factors that affect the distribution, making the results more comprehensive and reliable.

### Data preprocessing

#### Data preprocessing for residential buildings

Since the simulation of population distribution is carried out in the unit of residential buildings, the data of residential buildings in Qingdao need to be obtained first. The existing imagery data were reclassified and the city of Qingdao was selected as the research object. We Intersected the residential land range with the building vector data to obtain the residential building data. Because there are still a few fine map spots (such as carport, distribution box, etc.) within the residential area, we used the map spot area, perimeter and other conditions and visual interpretation to remove invalid map spots, and then complete residential building data in Qingdao can be obtained. However, it should be noted that residential land does not include school dormitories, factory dormitories, etc., so this paper will not include such buildings in population simulation.

#### Generating building height

By reclassification, we can get the site area of the residential building. But a lot of buildings are not bungalows, so we also need to capture the height of the buildings.

Due to the high floor and density of buildings in urban areas, it is difficult to carry out rapid field measurement of large areas. We use DSM data and DEM data to extract the height of urban buildings [33, 34]. The height is defined as the difference between the elevation of the top of the building and the elevation of the DEM at the bottom of the building, as shown in (1).


height=DSM−DEM (1)

#### Luojia 1–01 NTL data preprocessing

Due to the wide area of Qingdao, it is necessary to splice several adjacent remote sensing imageries into a large-scale seamless imagery. Then we chose the image mapping method, using the main road remote sensing imagery of Qingdao City, manually selected 30 uniform road intersections as ground control points (GCPs), carry out accurate geometric correction on the original data of Qingdao NTL remote sensing imagery, and cut out the NTL data in Qingdao administrative boundary area. The negative value of pixels contained in the original NTL data is considered to be the noise value generated in the process of data synthesis [35]. In order to ensure the integrity of the data, the negative value is replaced with 0 for processing.

According to [23], the Luojia 1–01 NTL imagery need to perform radiometric correction. The digital number (DN) value was converted into the radiant brightness (RB) value to participate in the subsequent calculation. The radiance conversion formula of Luojia 1–01 NTL is as follows:

$$L=10^{‐10}\times DN^{\frac{3}{2}}$$ (2)

where *L* represents the RB values with the unit W/(m^2^⋅sr⋅μm),and *DN* represents the DN values for a pixel.

#### Generating a HSI image

**Fig 3:**
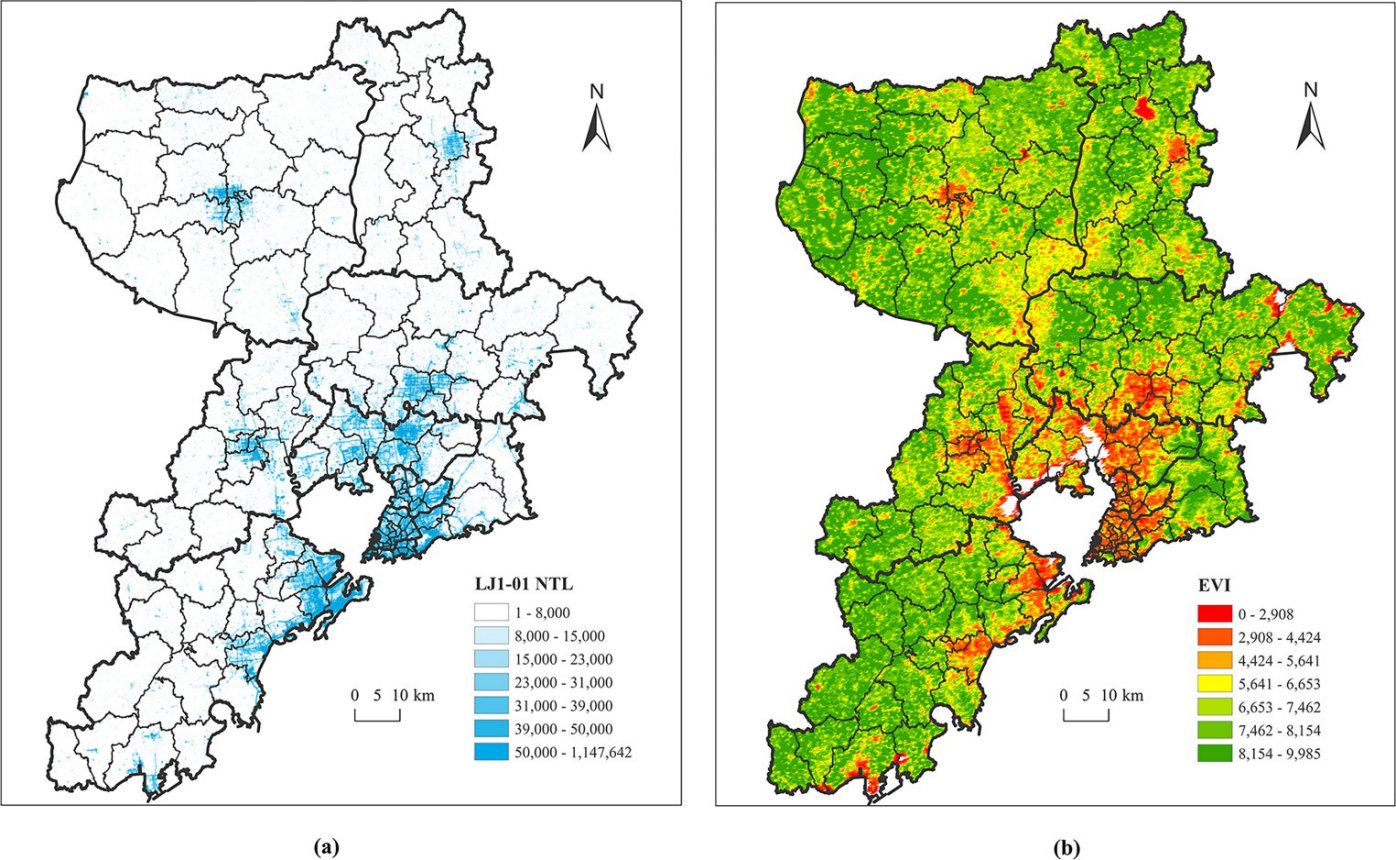
(A) The Luojia 1–01 NTL imagery of Qingdao in 2018; (b) The EVI imagery of Qingdao in 2018. We used the bilinear interpolation algorithm to unify the spatial resolution of Luojia 1–01 NTL data (Fig 3A) and EVI data (Fig 3B) to 100m, and calculated HSI values for Qingdao City in 2018 by using (3) [32].


Fig 3 shows the spatial distribution of Luojia 1–01 NTL and annual maximum EVI value in Qingdao in 2018. Both of them have a good spatial correspondence and are closely related to human activities. In order to eliminate the influence of cloud in EVI, we generated new EVI composite images by selecting the maximum value of each pixel from the 9 EVI images taken in 2018.

$$\mathrm{HSI}=\frac{1-EVI_{max}+L_{nor}}{1-L_{nor}+EVI_{max}+L_{nor}\times EVI_{max}}$$ (3)

where *L*_*nor*_ denotes the normalized RB values of the Luojia 1–01 NTL imagery, which was calculated as follows:

$$L_{nor}=\frac{L-L_{min}}{L_{max}-L_{min}}$$ (4)

where *L*_*max*_ and *L*_*min*_, respectively, denote the maximum and minimum RB values of the Luojia 1–01 NTL imagery in Qingdao.

#### POI data preprocessing

In order to obtain the socio-economic factors affecting population distribution near residential buildings, we chose POI big data as the simulated social impact factor. Since the overall trend of spatial distribution of NTL and POI is consistent, some studies fuse POI with NTL to identify urban central areas after kernel density analysis. Therefore, we also chose to carry out kernel density analysis on each type of POI, and the results reflect that residential buildings are affected by various industries [36–38]. Kernel density analysis was carried out on the cleaned POI data according to classification, and a kernel density map of 100m×100m was constructed respectively.

#### Residential attribute mapping

**Fig 4:**
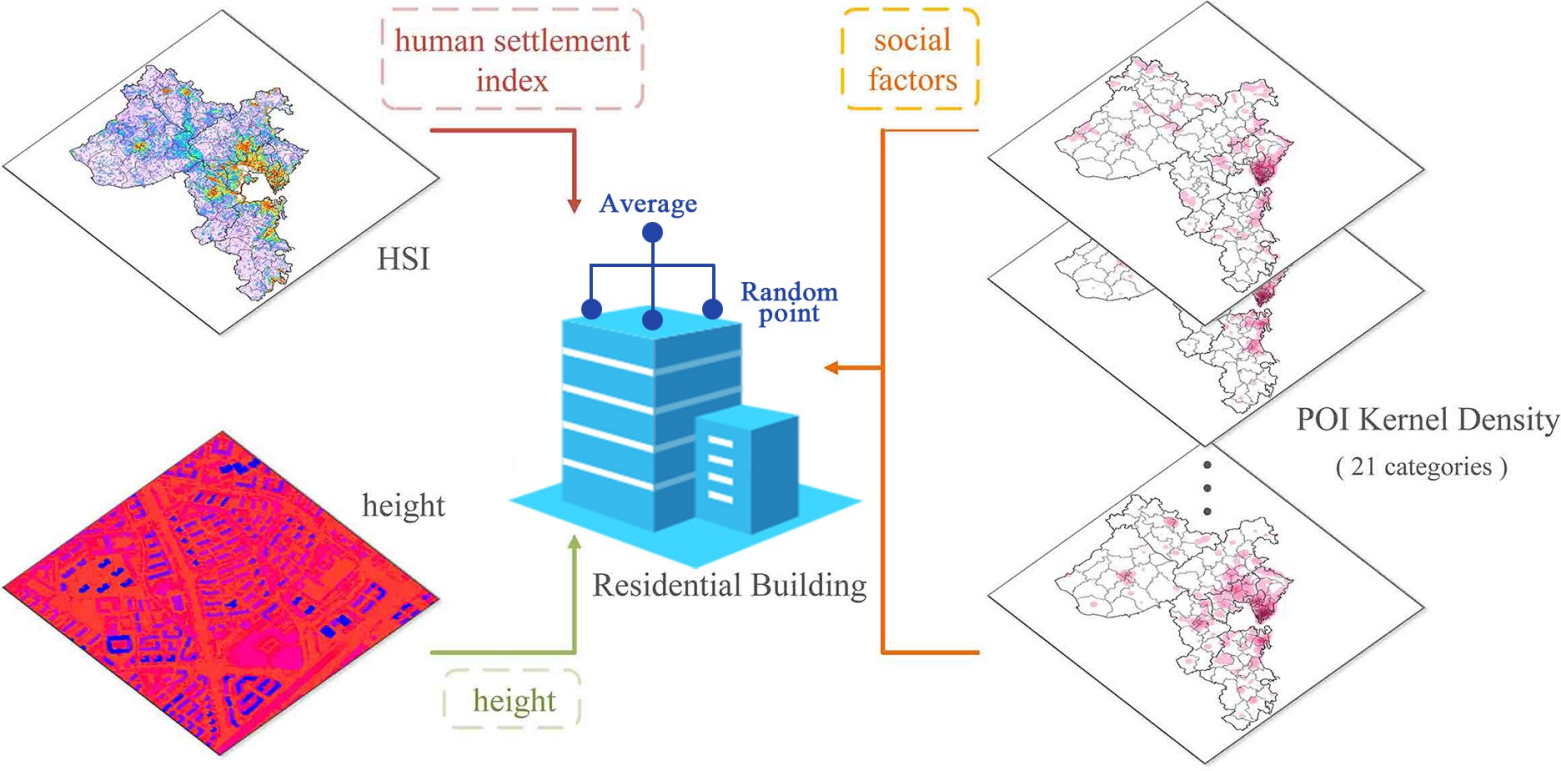
Attribute mapping of residential buildings.

We need to map the height, the HSI and the POI kernel density to residential building data respectively before constructing a population distribution model. Because these three kinds of data need to be mapped are all raster data, the real residential building may not be exactly in a grid, it may span multiple grids at the same time. Therefore, we took random points inside residential buildings and calculate the average value of all sampling points as the attributes (height, HSI, and social factors) of each residential building to establish the relationship between residential buildings and multi-source urban geographic data (Fig 4).

### The construction of RPI

In order to reflect the relationship between residential building floor area and resident population, we put forward a new index called RPI. In this study, RPI is constructed to explain the relationship between total floor area of residential building and the residential population number, and the calculation formula is

$$\mathrm{RPI}=\frac{P_{RB}}{S_{RB}}$$ (5)

Where *P*_*RB*_ represents the number of the population in the residence, and *S*_*RB*_ represents the living area of the residence. Among them, the calculation formula of residential living area *S*_*RB*_ is:

$$S_{RB}=A_{RB}\times \frac{H_{RB}}{h}$$ (6)

Where *A*_*RB*_ represents the base area of residential buildings, *H*_*RB*_ represents the height of residential buildings, and *h* represents the average floor height of residential buildings. According to "the State Council about the regulation that strictly controls the standard of town residence" in the regulation, residential floor height presses 2.8m calculation.

After calculating the total area of each residential building by using (5), the total residential area of each township and street is counted. And according to the resident population statistics data of each district in *Qingdao Yearbook 2019*, the RPI_*str*_ of each township street is estimated. Because residential buildings do not contain school dormitories, in order to more accurately simulate the distribution of permanent resident population, the number of school population should be removed from the resident population statistics data of each district where colleges and universities are located. Although some factories also have dormitories, due to the complex situation of factory workers, including not only those who live in dormitories, those who rent houses outside, but also temporary workers, it is not easy to calculate and has little influence on the final result, so it is ignored.

### Screening POI indicators

Since POI data are divided into 21 categories, and not every category of data is related to RPI, it is necessary to first calculate the average kernel density of towns and streets of each category of POI and conduct correlation test with RPI_*str*_ after removing an outlier. The spearman correlation coefficient is shown.

**Fig 5:**
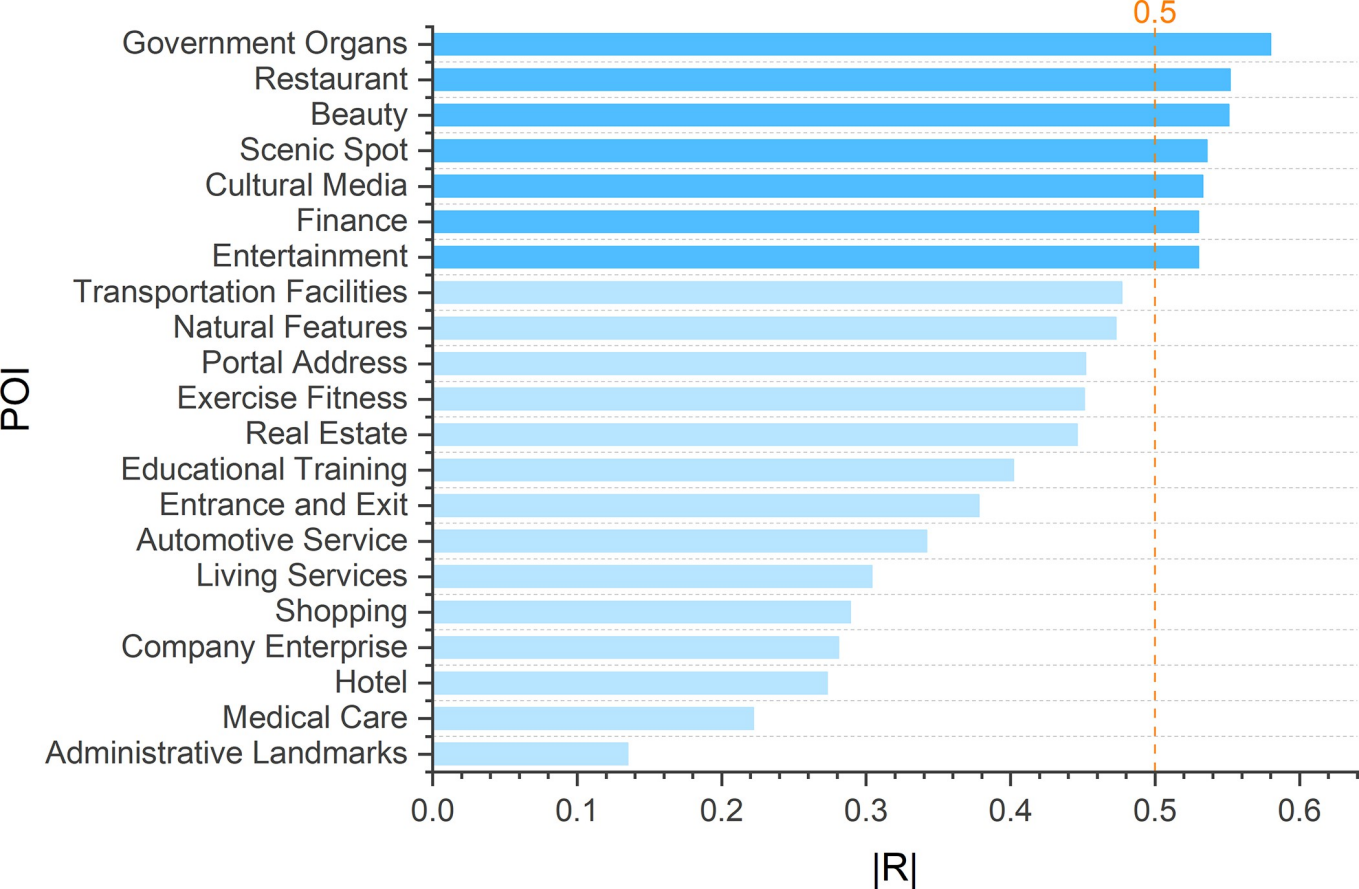
Absolute value of spearman correlation coefficient between the mean kernel density and RPI_*str*_ of township streets of 21 POIs. Correlation is significant at the 0.01 level (2-tailed).

As can be seen from Fig 5, the absolute value of correlation coefficient between street average kernel density and RPI_*str*_ of 7 types of POI is above 0.5. Therefore, these 7 types of POI are selected as social factor indicators affecting RPI [6, 39, 40].

### The extraction of principal components

**Fig 6:**
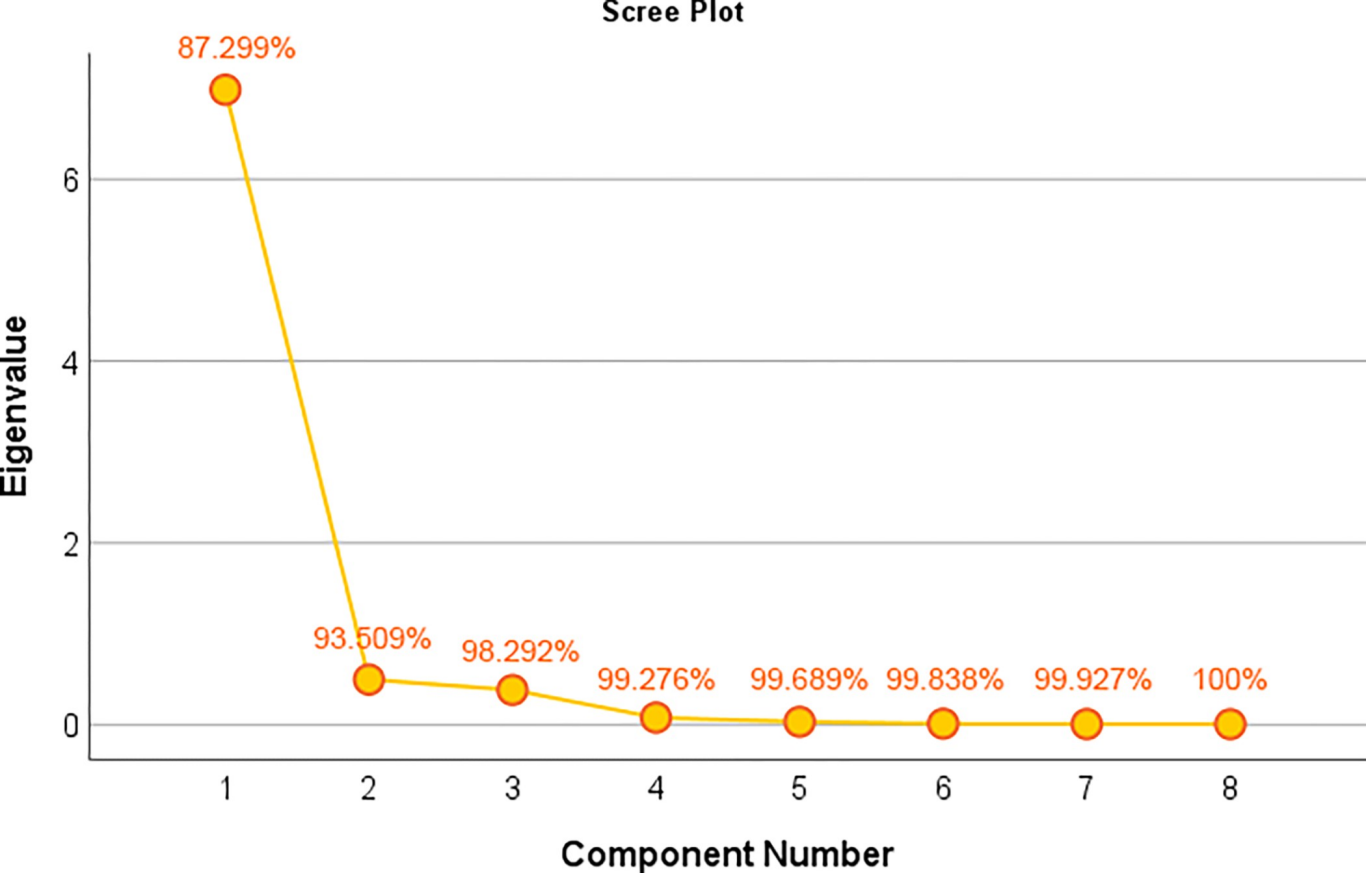
Broken stone diagram of principal components after dimensionality reduction.

Through calculation, the absolute value of correlation coefficient between HSI and RPI_*str*_ is also greater than 0.5, so we can assume that HSI and 7 types of POI are all correlated with RPI. But there is a high correlation between these indicators, and direct establishment of regression model may lead to instability. Therefore, we first use principal component analysis to reduce the correlation between indicators, and integrate multiple indicators into a few independent principal components, so as to avoid the impact of multicollinearity. The average HSI of streets was counted and the average kernel density of streets of HSI and 7 kinds of POI was tested by KMO test, which used to compare simple and partial correlation coefficients between variables. The closer the KMO test statistic is to 1, the stronger the correlation between variables is. This KMO test statistic was 0.847 and the significance was 0, indicating that the indicators were strongly correlated and suitable for principal component analysis. The broken stone diagram can show how much variable information the factor covers, and its vertical axis represents the size of the characteristic root of the factor. As Fig 6 shows, after the second characteristic root, the cumulative variance contribution rate has reached 93.509%. Therefore, the first two characteristic roots are selected as the main factors.

## Results

### PCR-based RPI prediction

The two common factors obtained from the principal component analysis were combined with RPI_*str*_ to establish RPI_*str*_ standardized principal component regression equation, as shown in (7).


$$Y=0.687\times F_{1}+0.181\times F_{2}$$ (7)

**Fig 7:**
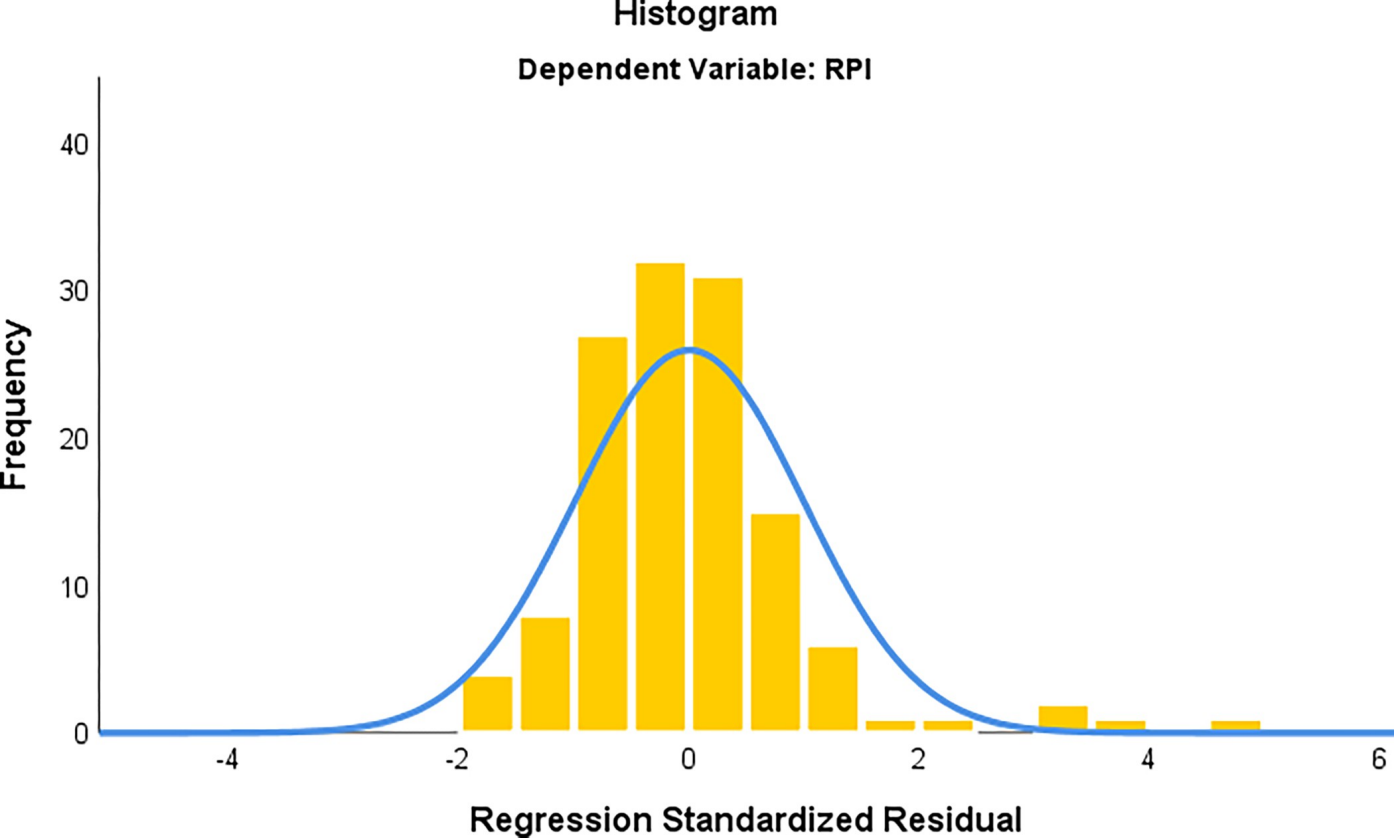
Distribution diagram of principal component regression prediction model.

Where *F*_1_ and *F*_2_ are the two principal components extracted. The value obtained by Durbin-Watson test is 1.942. Since the closer DW value is to 2, the less the residual terms are correlated, it can be said that this regression model is unbiased. The significance is lower than 0.01, and the VIF is 1, indicating that multicollinearity has been eliminated. According to the Fig 7, residuals conform to normal distribution, indicating that this model is effective in predicting RPI.

The transformed RPI prediction model is:

$$\begin{array}{l} \mathrm{RPI}=5.93E-05\times X_{1}+4.6E-05\times X_{2}+4.95E-04\times X_{3}+3.46E-05\times X_{4}+2.53E-04\times X_{5}\\ \;+1.65E-04\times X_{6}+1.54E-04\times X_{7}+1.05E-02\times X_{8}-4.3E-03 \end{array}$$ (8)

Where *X*_1_~*X*_8_ represent 8 indicators of finance, beauty, scenic spot, restaurant, cultural media, entertainment, government organs, NTL respectively. After the prediction model is obtained, the RPI of each residence can be predicted by substituting residential building data into (8).

### Simulated population distribution

Since the RPI of each residence can be predicted by integrating NTL and multi-source urban geographical data, the estimated population in the residence can be deduced by substituting the predicted residential RPI and residential floor area into the (9), and the population distribution map of Qingdao City was drawn on this basis.


$$POP^{est}=\mathrm{RPI}\times S_{RB}$$ (9)

**Fig 8:**
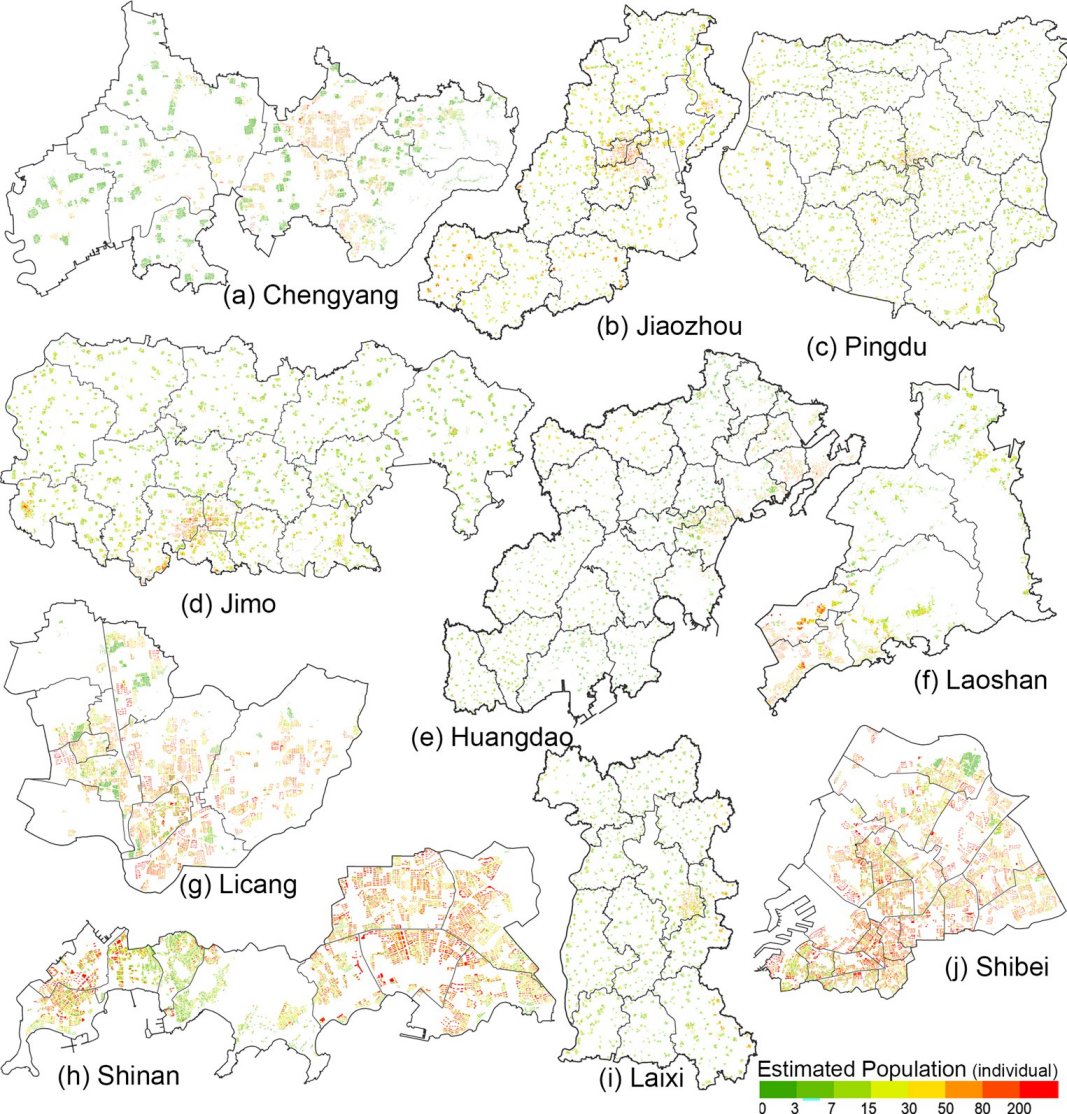
Resident population in Qingdao in 2018.

Fig 8 shows the distribution of resident population based on the model calculation. Compared with the traditional population density map based on district division, the results obtained are more representative. Take Laoshan District as an example (Fig 8F). Laoshan Mountain, located in the east of Laoshan District of Qingdao City, is the main mountain range on the Shandong Peninsula. Although the land area of Laoshan District is 395.79 square kilometers, most of its jurisdiction is occupied by Laoshan Mountains. Therefore, although the area is vast, the inhabitable area is small. In addition, as Laoshan District is a well-developed administrative region in recent years, most of the residential buildings are high-rise residential buildings. In the past, the results of simulated population distribution based on LULC would suggest that the population density in this area is low and population distribution is not strongly influenced by mountains, but this is not the case. It can be found in Fig 8F that there are non-residential areas in Laoshan Mountains, and only some villages along the coast of Laoshan are inhabited. Moreover, the urban area of Laoshan District also conforms to the characteristics of population distribution of high-rise residential areas. We simulated population distribution based on real residential buildings, which not only retained the influence of Laoshan Mountain on population distribution, but also reflected the real residential situation.

From the perspective of population distribution trend, the residence with more population is basically in the region with higher brightness displayed by NTL. The residential buildings with large population are mainly concentrated in the coastal areas of Jiaozhou Bay, especially in Shinan district (Fig 8H) and Shibei district (Fig 8J). On the other hand, areas with relatively low urbanization degree, such as Jimo city (Fig 8D) and Pingdu City (Fig 8C), have a small number of the resident population in the residences. However, due to the vast area of these areas, the total population is not low. The results are consistent with the actual situation. Therefore, this method can not only clearly reflect the distribution of urban resident population, but also explain more details, which can be used as an important database for urbanization work.

### Accuracy assessment

#### Field sampling survey

**Fig 9:**
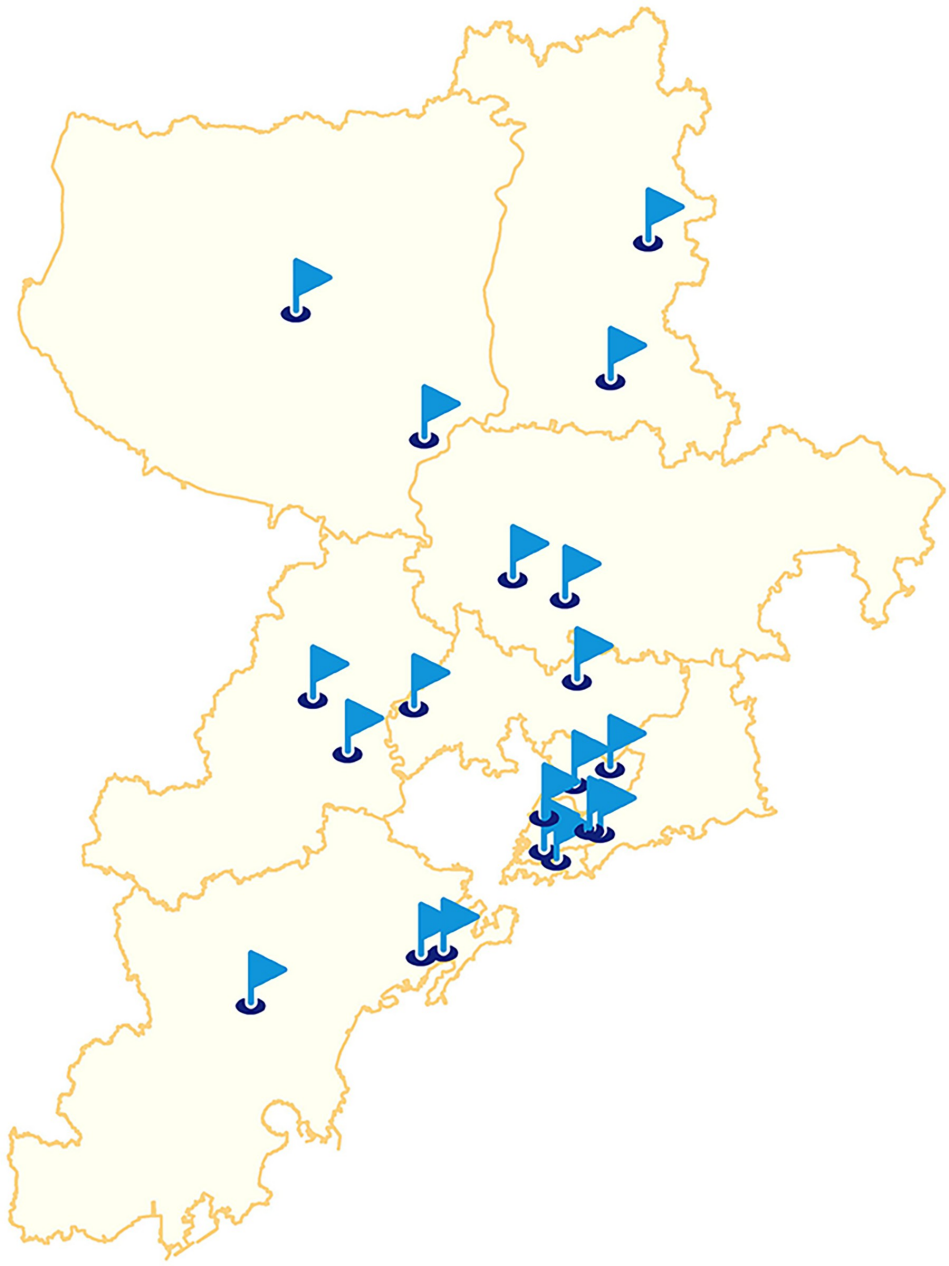
The location distribution map of 20 residential communities selected as a random sample.


In order to verify the reliability of the results, we randomly selected 20 residential communities from 10 administrative regions for field investigation. The location of random sampling residences is shown in the Fig 9. The MRE between the predicted results and the field survey results is about 14.5%. The MRE is used to measure the deviation degree between the estimated value of the model and the true value. The smaller the MRE, the higher the reliability of the estimated result of the model.

$$\mathrm{MRE}=\frac{1}{n}\sum_{i=1}^{n}\frac{\left|POP_{i}^{est}-POP_{i}\right|}{POP_{i}}$$ (10)

where $POP_{i}^{est}$ represents the estimated population of the ith town, *POP*_*i*_ represents the actual population of the ith town, *n* is the number of towns.

**Fig 10:**
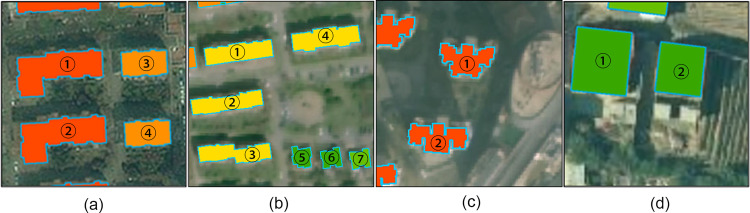
(A) ① and ② have 6 floors and a loft. ③ and ④ have 6 floors; (b) ① and ④ have 5 floors, ② and ③ have 6 floors, and ⑤ to ⑦ are single-family villas; (c) ① has 19 floors, and ② has 18 floors; (d) ① and ② are both rural buildings [31].

We have selected four different types of residential communities and shown them in detail in Fig 10.

In Fig 10A, built in 1993, this community is located in a prosperous area with rich educational and medical resources and convenient transportation. The occupancy rate of the community is extremely high, and most of the residents are middle-aged elderly. The estimated population in ① to ④ is 111, 129, 57 and 58 respectively, with an average error of 8.75%; In Fig 10B, built in 2001, this community has a quiet environment and moderate occupancy rate. The main housing area of the community is about 100 square meters, and most of the residents are in the nearby work or school families. The estimated population in ① to ⑦ is 35, 46, 46, 38, 3, 3, and 4 respectively, with an average error of 16.2%; In Fig 10C, the community was built in 2009 in a busy business district and close to the city’s main roads. The main housing area of the community is about 130 square meters. Due to its large housing area, most of the residents are families with three generations living together. The estimated population in ① to ② is 166 and 174 respectively, with an average error of 10.25%; In Fig 10D, ① and ② are both rural self-built residence located at the edge of a field. The estimated population in ① to ② is 3 and 3 respectively, with an average error of 0.

#### Overall assessment

**Fig 11:**
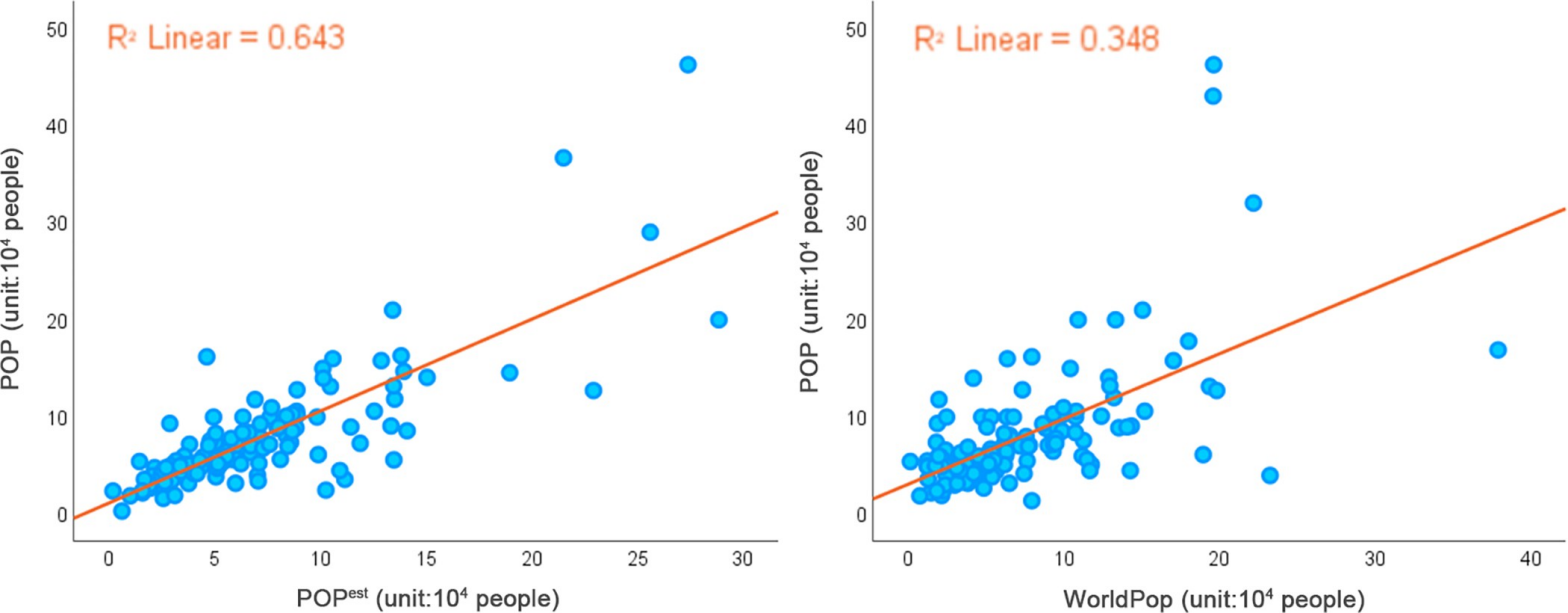
R^2^ between (a) *POP*^*est*^ and (b) Worldpop datasets and real statistical results.

At present, mainstream research uses the method of verifying the spatial population data by administrative division. The mean relative error (MRE), root mean square error (RMSE), relative root mean square error (%RMSE) and coefficient of determination (R^2^) were obtained to measure the accuracy of population grid data [33]. We conducted further tests on the fitting of the estimated results according to *Qingdao Yearbook 2019*. The estimated population was counted by the unit of township streets and compared with the real statistical results to calculate the coefficient of determination (Fig 11). At the same time, in order to evaluate the fitting accuracy, the influential 2018 Worldpop population distribution dataset was used for comparison.

To emphasize that this research model can improve the accuracy of the population density figure, we also calculated the mean relative error (MRE) and the root mean square error divided by the average street population (%RMSE). The %RMSE is the ratio of the square between the estimated value and the true value to the total sample, which is considered to be used to judge the performance of the algorithm and reflect the accuracy of the results. These metrics are calculated as:

$$\%\mathrm{RMSE}=\frac{\sqrt{\frac{\sum_{i=1}^{n}{\left(POP_{i}^{est}-POP_{i}\right)}^{2}}{n}}}{M_{pop}}$$ (11)

where $POP_{i}^{est}$ represents the estimated population of the ith town, *POP*_*i*_ represents the actual population of the ith town, *n* is the number of towns, and *M*_*pop*_ is the mean township population.

Table 2 summarized the accuracy verification results of *POP*^*est*^ and Worldpop datasets. The MRE and %RMSE of Worldpop dataset were 44.02 and 67.17%, respectively. while the MRE (33.6) and %RMSE (43.65%) of *POP*^*est*^ predicted by this method were significantly decreased, which was significantly lower than that of Worldpop dataset. Meanwhile, R^2^ ranges from 0 to 1, and the closer it is to 1, the stronger the correlation is. Therefore, it can be concluded that the method adopted in this paper can effectively improve the accuracy of simulating population distribution.

**Table 2:** Comparison of errors between *POP*^*est*^ and Worldpop and real statistical data.

	WorldPop	*POP* ^*est*^
**R^2^**	0.348	0.643
**MRE (%)**	44.02	33.6
**%RMSE**	67.17	43.65

R^2^, coefficient of determination; MRE, the mean relative error; %RMSE, the root mean square error divided by the average street population.

**Fig 12:**
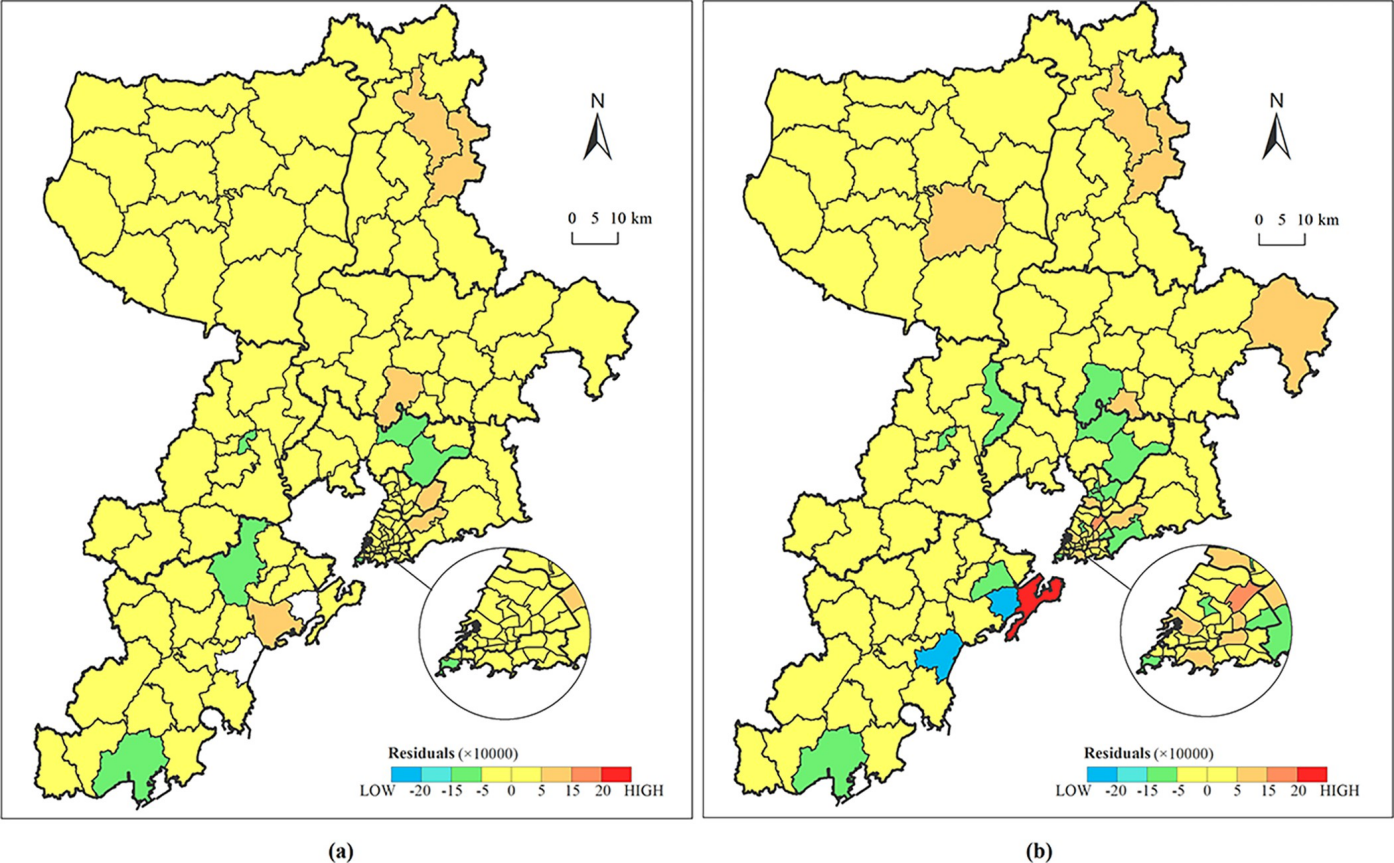
Residual distribution of estimated population between (a) *POP*^*est*^ and (b) Worldpop datasets and real statistics.

For a more intuitive representation, we compared the residual distribution of estimated population between *POP*^*est*^, Worldpop data sets and real statistical data. The residuals were obtained by subtracting the estimated population from the township street demographics. The red bars indicate overestimates of the population and the blue bars indicate underestimates of the population. The darker the color, the higher the overestimates or underestimates are (Fig 12).

The population prediction based on the integration of NTL and multi-source urban geographic data slightly overestimated or underestimated the population in a few areas, but has improved the residual distribution in general, especially for the Jiaozhou Bay, where the error was significantly lower than that of Worldpop data set. Meanwhile, in the Worldpop data set, the phenomenon of overestimation and underestimation is quite obvious, especially for Xuejiadao Street, Changjiang Road Street and Yinzhu Street. Therefore, multi-source urban geographic data can reduce the errors of population density modeling. For 17 township streets, however, there still exists obvious population forecasting error, which might be due to the impact of the floating population. In the future, for cities with high population mobility like Qingdao, more components can be integrated to optimize the model.

Based on the estimated population of residential buildings in the built-up area, the estimated urbanization rate is 69.7%, only 3.97% different from the data published by the Bureau of Statistics. Also compared with the Worldpop data set, the urbanization rate calculated by the statistics of built-up areas in the Worldpop data set is only 49.88%, which is nearly 24% different from the published data. Therefore, for cities with a large gap between urban and rural population and highly dense urban population, the method proposed in this paper can reasonably estimate the population according to the structure of different residential buildings, and the result is highly consistent with the urbanization rate.

## Discussion

China has been conducting population censuses for decades, but only seven of them have been carried out because of the huge amount of manpower and resources involved. In order to better assist the census work and obtain more accurate population distribution data with the advantages of low cost and high efficiency, this study proposed a residential population spatialization method based on multi-source urban geographic data. In previous studies, the population distribution density map is usually generated in grid format, which is generally used to reflect the overall distribution trend. Due to the smooth transition between grids and the lack of detail, there are certain limitations when it is used as basic urban data. This study takes residential buildings as the unit to study population distribution and proposes RPI, which takes into account the height of buildings rather than just relying on the residential base to simulate population distribution, reflects the relationship between total residential floor area and resident population. Since China is in the stage of rapid development of urbanization, a lot of high-rise residential buildings has sprung up, but there are still a lot of low rise rural residences in the edge of the city. Even with the same base area, the total floor area will be different depending on the height of the residential buildings. In this case, RPI can adapt to a variety of residential building structures and improve the heterogeneity and complexity of population distribution.

In addition to proposing new indicators, this study also integrated multi-source urban geographic data to improve the method of population spatialization, comprehensively considers a variety of environmental factors, and adjusted residential situation through geographically weighted indicators related to population distribution. This paper can present fine population distribution results comparable to the census results, reflect the distribution of resident population at specific residences, and enhance spatial heterogeneity. DSM and DEM data can provide the height information of residential buildings to simulate the distribution of population in vertical space. Because of the correlation between NTL, EVI and population distribution, the distribution of population can be effectively simulated. POIs data related to human activities can be used as various socioeconomic factors affecting residential population distribution, making the forecast results more comprehensive. In this paper, principal component regression method is used to get the relationship between various indicators and residential RPI, so as to reflect the number of residential people in the residence, the model is highly reliable and universal. NTL and EVI belong to natural remote sensing, while POIs is big data from social perception. By combining the two data, this method not only has the advantages of high timeliness, high stability, high speed and high economic benefit of remote sensing image, but also has the advantages of diverse data types and large volume.

In order to verify the correctness of the population distribution data of Qingdao city in 2018 calculated in this study, we adopted two methods: field sampling survey and overall assessment. First, through random sampling, we selected 20 samples of different types, scattered in each administrative district of Qingdao city for field investigation. Overall, the results were roughly in line with reality, with an average error of 14.5%. Second, we calculated the forecasted population by township street and compared the demographic data with the R^2^, the MRE and % RMSE. In order to reflect the optimization degree of the method, we also selected the Worldpop data set for comparison. The results show that, compared with the Worldpop dataset, the complex correlation coefficient is significantly increased, and the MRE and % RMSE are decreased, which can provide a more reliable resident population distribution.

Although the residential population spatialization method based on multi-source urban geographic data has certain practical significance, there are still the following shortcomings:

Because the land type where the university is located is generally educational land, and the teaching building and dormitory cannot be distinguished. In order to better simulate the distribution of permanent residents in most residential buildings and make the method more universal, this paper selectively ignores the permanent residents with special living situations. If accurate dormitory data is available, the results can be further supplemented.

This study used the data in Qingdao yearbook 2019 about township street population statistics to verify the accuracy of the result of population spatialization. But it’s still difficult to reflect the precision of the results on micro scale. Although this study has also carried out field verification, the amount of residential building data is too large and the random sample is too small. There are some defects, mainly lead by the difficultly to obtain statistics of administrative unit population on fine scales. In the future research, more fine-scale population verification data should be collected to establish a more complete accuracy verification system.

## Supporting information

S1 Data Estimated population of towns and streets in Qingdao in 2018. (XLS)
